# Physical Diagnosis*Abstract of a lecture delivered at Columbia Veterinary College.

**Published:** 1883-10

**Authors:** E. Benjamin Ramsdell

**Affiliations:** Lecturer on Diseases of the Respiratory Apparatus, Columbia Veterinary College and School of Comparative Medicine


### Art. XXVI.—PHYSICAL DIAGNOSIS.*

BY E. BENJAMIN RAMSDELL, M.D.

*Lecturer on Diseases of the Respiratory Apparatus, Columbia Veterinary College and School of Comparative Medicine.*

The subject of our lecture to-day will be auscultation, the most important of all the methods employed in physical diagnosis. Auscultation is that process by which we listen to sounds that are produced in parts hidden from our eyes, where we cannot approach to examine without a sudden and complete cessation of their movements and thereby of their functions.

Like in percussion, we have two distinct varieties; first, the immediate; and secondly, the mediate auscultation. In the immediate variety we place the ear directly upon the chest wall, which should be covered with a thin sheet. Mediate auscultation is where we interpose between our ear and the chest wall an instrument called a stethoscope, which not only

*Abstract of a lecture delivered at Columbia Veterinary College.

intensifies the intrathoracic sounds, but also excludes all foreign noises. In my hand I hold two varieties of the stethoscope; one, the straight monaural stethoscope of Lænnec; the other, the so-called Camman's stethoscope which has two arms which terminating in tips that enter both ears.

The binaural instrument is by far the better for many reasons which can be appreciated by all. It is well for us to become accustomed to both methods of auscultation and each one should provide himself with a binaural stethoscope. In the study of the sounds produced by the respiratory apparatus we have not *one* normal sound as we noted when studying percussion; but we have *several* sounds which are normal when heard in certain places although decidedly *abnormal* when heard in other localities.

The most important of the normal sounds is the vesicular murmur, as it is called, which comes to our ear when we have healthy lung tissue between the chest wall and the bronchial tubes. Flint, in his Physiology, states that this normal vesicular murmur is produced "chiefly by the expansion of the innumerable air cells of the lungs." However, *expansion* is not as fertile a source of sound as *vibration*, and I believe that this sound is simply a modification of the sound produced by the vibration of air in the first, second and third divisions of the bronchial tubes. This sound is conveyed to our ear by a layer of air vesicles, which are little pockets of air surrounded by tense walls. This poorly conducting layer so modifies the sound that when it comes to our ear it has acquired those properties by which we designate the normal vesicular murmur. Again there is no direct current of air in the minute bronchi or in the air vesicles, for the air that enters with every inspiration being only one-eleventh the amount in the lungs, advances only as far as the second divisions of the bronchial tubes; the changes in the air further on take place by the principle of diffusion of gases which has been explained in my lectures on Physics.

This vesicular mumur has two portions, an inspiratory and and expiratory part. This fact you may study by observing that the inspiratory sound is produced as the chest wall moves outward. This will be a guide to you until you become thoroughly acquainted with the pulmonary sounds.

The remaining part of the murmur is the expiratory portion, although there is no stop between the parts. It is one continuous sound; the only observable distinction is that the two parts differ a little in pitch. The expiratory part of the vesicular murmur is very much shorter, its pitch higher and its intensity less than that of the inspiratory portion.

This vesicular murmur, you will recollect, can only be heard when we have *healthy* lung tissue beneath our ear or stethoscope; and even this has an exception. For instance, in a case of pleurisy with effusion, although the lung may be healthy at the lower portion of the chest, yet the fluid effusion which lies between the chest wall and the lung will prevent the murmur from reaching the surface; and our ear, when placed over the area of the liquid, hears no sound. It is necessary, therefore, that the lung not alone must be healthy, but also must be in contact with the chest wall in order that the vesicular murmur can be heard.

If you place the ear or stethoscope over the trachea you will get a tubular sound, of which the first or inspiratory part is higher in pitch and has more intensity than that of the vesicular murmur. This first, or inspiratory, part of the sound ends before the act of inspiration is completed, so that there is a slight, although clearly defined, pause between the inspiratory and expiratory sound which is higher in pitch and has a duration as long or even longer than the inspiratory portion.

If you listen over the antero-sternal space in the lower animals or the supra-sternal space in man you will hear the so-called bronchial respiration, which is a sound you should become very familiar with. It has many points of resemblance to the tracheal sound, although not so well marked either in quality or intensity; in other words we say that the tracheal sound is an exaggerated bronchial respiration.

In *health* this bronchial respiration can be heard only in this region in the horse, but in man and in some of the lower animals also, in the inter-scapular region.

In disease of the lungs this bronchial sound may be heard over any part of the thorax; due to the fact that when any portion of the lung tissue gets into a condition so that it can conduct sound to the chest wall *perfectly*, the sound produced in the bronchial tubes will be conveyed to the ear in its true condition without the modification that I claim occurs in health.

Solidified lung is a very much better conductor of sound than healthy lung tissue, so that no matter whether the air vesicles are filled with *serum*, *e. g.*, in œdema of the lungs, *blood*, *e. g.*, hemorrhagic infarction, or *inflammatory products*, *e. g.*, pneumonia; in each and every case we will get the bronchial respiration.

In certain conditions of the lungs we hear a sound which although similar to the normal vesicular murmur, yet is much increased in intensity and duration; this occurs where one lung is doing double duty on account of disease on the other side. It is called the exaggerated respiration. Diminished or feeble respiration, where the intensity and duration are very much lessened, is the result of pleurisy, rheumatism, or paralysis of intercostal muscles, or emphysema. Absent or suppressed respiration is caused by large pleuritic effusions, pneumo-thorax, or where there is a complete obstruction of a large bronchus.

After studying the normal sounds with their variations we now come to a class of respiratory sounds which are heard only in disease. These have been named adventitious sounds or rales. A rale is a sound heard only in disease and has characteristics entirely different from the normal sounds. It may be produced along with the normal murmurs or may take the place of them. It may originate in the trachea, in a large or small bronchus, in the capillary bronchi, in the air cells, or in abnormal cavities situated within the lung substance.

It may be produced within the bronchial tubes either by a diminution of their calibre, caused by thickening of their mucous lining, by the vibration of viscid matter collected in them, or by air bubbling through fluid, either blood, pus, serum, or mucus, situated either in the trachea, (large or small) bronchi, or in cavities in the lung tissue.

Rales may be heard either during inspiration or expiration, or during both. From their nature you can readily see how rational it is to divide them into dry and moist rales, according not alone to their character, but also on account of the manner of their production.

Under the heading "dry rales" we have two classes; first, the sonorous, and, secondly, the sibilant rales.

The sonorous rale is a low-pitched sound with a decidedly snoring character produced by the vibration of a column of air which is passing over a roughened or irregular surface. These sounds arise in the larger bronchial tubes when we have an inflammatory thickening of the mucous layer or else a spasm of the muscular layer causing a diminution of the calibre of the tube or a corrugation of its mucous lining.

The sibilant rales are produced under precisely the same conditions as the sonorous rales, except that the former arise only in the smallest bronchial tubes.

The sibilant rale is a dry rale with a hissing, whistling character, and is higher pitched than the sonorous rale.

Both classes of dry rales are heard during both inspiration and expiration.

These sounds are usually indicative of the first stage of bronchitis; that is an inflammation of the mucous membrane lining these tubes. The sonorous rale indicating inflammation in the large bronchi, while the sibilant rale points to an inflammation in the smallest bronchial tubes.

Of the moist rales we have four distinct varieties; first, crepitant rales; secondly, sub-crepitant rales; thirdly, mucous rales; and fourthly, gurgles.

The crepitant rales which are heard only during the latter part and at the end of inspiration have been likened to the fine crackling produced by pinching and rubbing a lock of hair between the thumb and forefinger immediately in front of the ear. Two theories as to its production have been advanced; first, that it is caused by the separating at the end of inspiration of the walls of the air vesicles, which have been glued together during expiration by a viscid exudation, the result of inflammation; secondly, that it is produced by minute bubbles of air passing through a fluid at the mouth of the air vesicles. This rale is indicative of the first stage of pneumonia, or of œdema of the lungs.

The sub-crepitant rale is heard both during inspiration and expiration, and sounds like minute bubbles. Such is really the case, for the sound is produced in the smallest bronchi by minute bubbles of air passing through fluid, either serum, blood, pus, or mucus.

Thus it is manifest in œdema of the lungs, in hemorrhage, in the moist stage of capillary bronchitis and in the last stage of pneumonia.

Mucous rales are nothing more than large or exaggerated subcrepitant rales. They are large, bubbling sound, heard both during inspiration and expiration and are produced in the bronchial tubes by bubbles of air passing through mucus, serum, pus, or blood. They are usually present during the moist stage of bronchitis, or in advanced œdema of the lungs, etc.

We have, of course, large and small mucous rales, or, as they are called by some diagnosticians, "coarse" and "fine" rales. These differ according to the size of the bronchial tubes in which they are produced. "Coarse" rales being produced in the largest bronchi, while the "fine" rales occur in the smallest tubes.

Gurgles, the last form of moist rales, differ in their origin very much from the other kinds before mentioned, inasmuch as the gurgles are produced in cavities in the lung tissue, the result of a phthisical process, or an abscess of the lung following an unresolved pneumonia.

Where we have a cavity half-filled with fluid, usually pus, and a bronchus entering this cavity below the level of the fluid we will get this gurgling sound.

One other intra-thoracic sound, which is of exceeding great importance that you should apprehend is the pleuritic friction sound.

This sound, standing a class by itself, differs from the other sounds before mentioned, in that it is produced not within the lung, but outside of it.

That beautiful, delicate serous membrane, the pleura, whose two surfaces play one upon the other, so smoothly, so noiselessly, and so painlessly in health, becomes, under the injurious action of inflammation, so roughened that, besides the pain, besides the pleuritic friction fremitus that comes to us by palpation, it gives forth at each inspiration also a sound, a grazing, rubbing sound, in the acute inflammation, and as some describe it, a grating and creaking sound in chronic pleurisy. These sounds, heard at each inspiration, occur just over the point of inflammation, and we speak of them as the localized pleuritic friction sounds.

